# Two-dimensional power Doppler-three-dimensional ultrasound imaging of a cesarean section dehiscence with utero-peritoneal fistula: a case report

**DOI:** 10.1186/1752-1947-3-42

**Published:** 2009-01-30

**Authors:** Pedro Royo, Manuel García Manero, Begoña Olartecoechea, Juan Luis Alcázar

**Affiliations:** 1 Department of Obstetrics and Gynecology, Clinica Universitaria de Navarra, Avenida Pio XII, 36, 31008 Pamplona, Spain

## Abstract

**Introduction:**

An imaging diagnosis after an iterative cesarean delivery is reviewed demonstrating a fine ultrasound-pathologic correlation.

**Case presentation:**

A 33-year-old woman (G3, P3) presented referring intense dysmenorrhea and intermenstrual spotting since her third cesarean delivery, 1 year before. A cesarean section dehiscence with utero-peritoneal fistula was diagnosed by transvaginal ultrasound.

**Conclusion:**

We can conclude that transvaginal two-dimensional power Doppler and three-dimensional ultrasound are highly accurate in detecting cesarean section dehiscence and uterine fistula.

## Introduction

The uterine fistula is a known and uncommon entity as a possible result of gynecological surgery or other pathologic conditions [1]. The lower segment type of cesarean section has increased the prevalence of these uterine fistulous processes [1,2]. An imaging diagnosis after an iterative cesarean delivery is reviewed demonstrating a fine ultrasound-pathologic correlation. Our objective is to report an unusual case of utero-peritoneal fistula in cesarean scar defect diagnosed by color Doppler hysterosonography and three-dimensional ultrasound.

## Case presentation

**Figure 1:**
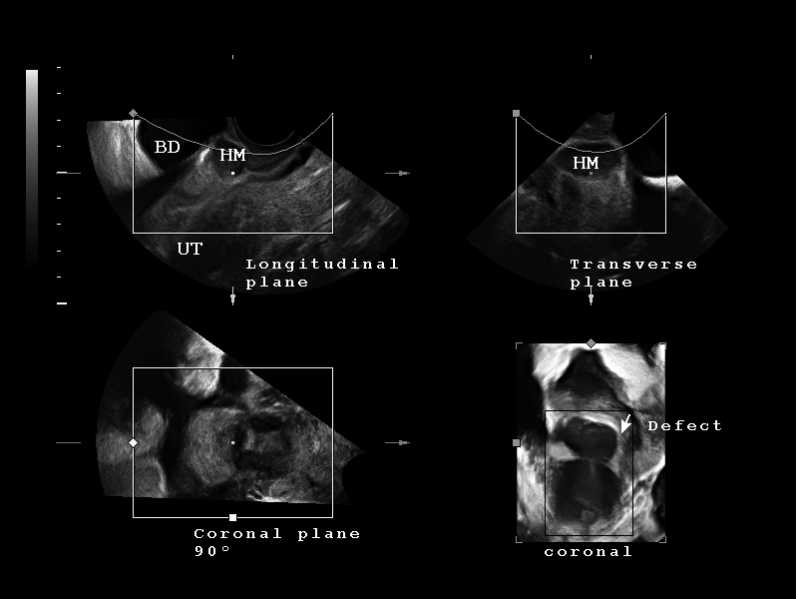
**Three-dimensional transvaginal ultrasound scan (in multiplane acquisition mode) of the uterus-hematoma-bladder complex (UT, HM, BD respectively)**. Please note that the white pixel (placed in the center of each image) always correspond with the same space point of the three orthogonal planes, and is located referring HM, between UT (at the level of the uterine scar) and just beneath BD. Defect's surface three-dimensional reconstruction (of the coronal plane) correspond with bottom right picture, and is framed with a white arrow (instead of white pixel).

**Figure 2:**
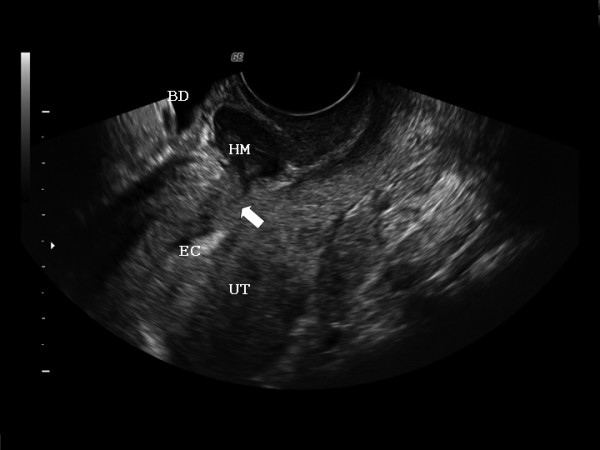
**Two-dimensional transvaginal uterine (UT) ultrasound on longitudinal plane showing the communication (arrow) of the hematoma (HM) with the endometrial cavity (EC)**.

**Figure 3:**
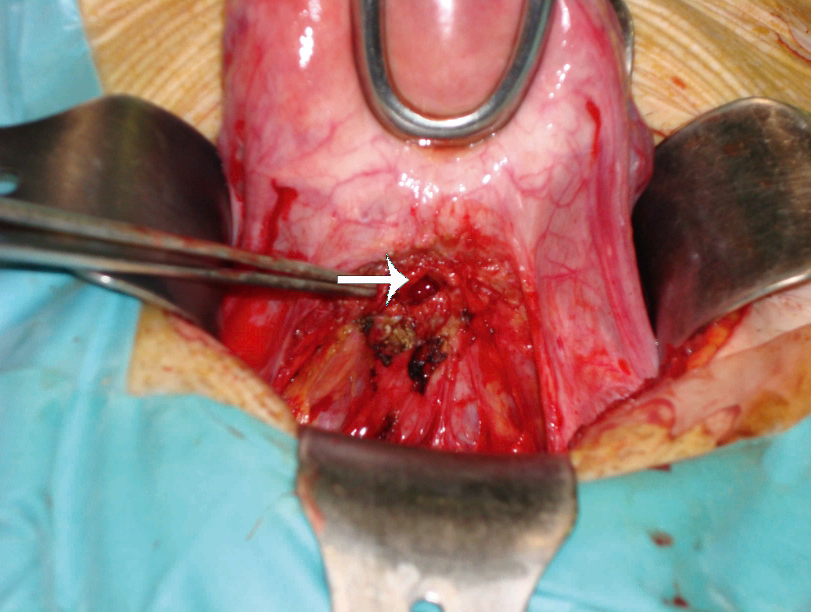
**Intra-operative picture showing the defect on the lower uterine segment after dissection (arrow)**.

**Figure 4:**
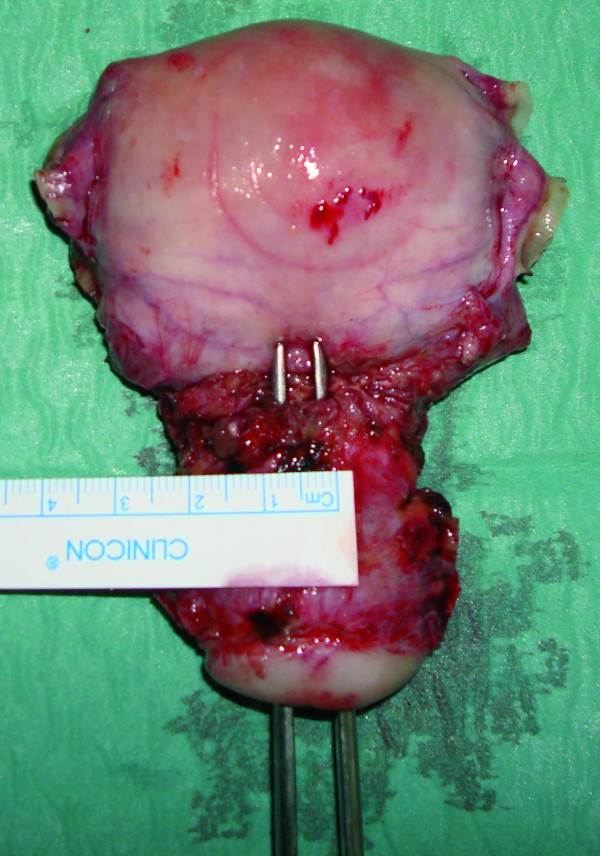
**Pathological image of the uterus showing the defect**.

A 33-year-old woman (G3, P3) presented referring intense dysmenorrhea and intermenstrual spotting since her third cesarean delivery, 1 year earlier. The patient's medical history and physical examination did not reveal any relevant finding. Two-dimensional-three-dimensional transvaginal ultrasound scans were performed with a Voluson 730 Expert system (GE Healthcare, Milwaukee, WI, USA) and IC5–9 (5–9 MHz) wide band Convex probe. Power Doppler settings were set to achieve maximum sensitivity to detect low velocity flow without noise (frequency, 5 MHz; power Doppler gain, -7.4; dynamic range, 20–40 dB; edge, 1; persistence, 2; color map, 5; gate, 2; filter, L1; and pulse repetition frequency, 0.6 kHz). The scan showed a hematoma (5.3 cm^3^) between the cesarean section scar and the bladder peritoneum. The bladder wall was not involved (Figure 1). The lower uterine segment had a 9 × 12 mm wall defect and an anechoic track that seemed to communicate the blood collection with the endometrial cavity (Figure 2). Afterwards, the power Doppler examination demonstrated the presence of active blood flow across the myometrium (Additional file 1). Finally, the treatment performed was an abdominal hysterectomy and the pathologic study confirmed the process as being of ischemic origin (Figures 3 and 4).

## Discussion

Uterine fistulas are infrequent pathologic entities and are characterized by abnormal communication of the uterus with any other organ or structure through a perforation due to traumatic or infectious conditions [1]. The lower segment type of cesarean section has increased the prevalence of these uterine fistulous processes, which account for 83% of cases [1,2]. Rarely, it could be related to long labor, forceps delivery, vaginal birth after cesarean section, gynecological injuries, tuberculosis of the genital tract or intrauterine contraceptive devices [2]. Our patient could not be considered as having Youseff's syndrome [3] because the bladder wall was not involved and, in addition, the three types of vesico-uterine fistulas defined by Jozwik and Jozwik were also ruled out [4]. This case must be considered as an utero-peritoneal fistula, because the uterovesical pouch of peritoneum that covers the ventral surface of the uterus (separated from the bladder) was not affected.

The presence of the fistula can explain the symptoms referred by the patient during her menstrual cycle, with the passage of blood to the peritoneal cavity (causing peritoneal irritation with pelvic pain) and the vagina (causing intermenstrual spotting) [1]. Transvaginal ultrasound and color Doppler hysterosonography have been used successfully in many cases to allow direct visualization of the uterine fistulae. It has been demonstrated that the normal sonographic appearance of the uterine incision as distinguishable from the abnormal appearance in patients who were symptomatic after cesarean section [5]. Benacerraf *et al*. [5] showed three sonographic patterns for the uterine scar, including a dense, echogenic area; a fluid-filled area anterior to the site of the wound between the uterus and the bladder (our case); and a sonolucent area at the site of the wound between the external surface of the lower uterine segment and the lumen of the uterus. Transvaginal ultrasound is highly accurate in detecting cesarean hysterotomy scars. The cesarean scar defect, defined by the presence of fluid within the incision site, is more common when labor precedes cesarean delivery and with multiple cesarean deliveries [1].

The advantage of three-dimensional gynecological ultrasound (Figure 1) is the possibility of obtaining coronal planes and their surface reconstruction which provides new image features which are not possible to obtain with conventional two-dimensional ultrasound [6].

As non-invasive alternative procedures, magnetic resonance imaging with heavily T2-weighted images may show a bright fluid-filled tract, and computed tomography can also be diagnostic [1,2,7].

Conservative management may be attempted, especially for patients with few symptoms, as the tract may spontaneously close [7,8]. The pregnancy rate after repair is 31.25% with a rate of term deliveries of 25% [2]. After dehiscence repair, due to the high risk of uterine rupture or dehiscence, a new delivery should be performed by repeating a cesarean section [2,7,8].

## Conclusion

Transvaginal two-dimensional power Doppler and three-dimensional ultrasound are highly accurate in detecting cesarean section dehiscence and uterine fistula.

## Consent

Written informed consent was obtained from the patient for publication of this case report and any accompanying images. A copy of the written consent is available for review by the Editor-in-Chief of this journal.

## Competing interests

The authors declare that they have no competing interests.

## Authors' contributions

PR (as corresponding author) and BO took intraoperatory photos, reviewed the literature and drafted the case description and discussion. MGM, a specialist in obstetrics and gynecology, revised and corrected all areas in the text covering this field. JLA, a specialist in obstetric and gynecology imaging, acquired and interpreted the sonographic images and revised and corrected all relevant areas of the text.

## Supplementary Material

Additional File 1 **Video**. Real-time B-mode and power Doppler video showing the blood moving between the hematoma and the endometrial cavity and which demonstrates the utero-peritoneal fistula. Click here for file
